# PReS13-SPK-1469: Juvenile scleroderma

**DOI:** 10.1186/1546-0096-11-S2-I19

**Published:** 2013-12-05

**Authors:** F Zulian

**Affiliations:** 1 University of Padua, Padova, Italy

## 

Juvenile localized scleroderma, also known as morphea, is the more frequent subtype of scleroderma in childhood. It comprises a group of distinct conditions which involve the skin and subcutaneous tissues. They range from very small plaques of fibrosis involving only the skin, to diseases which may cause significant functional and cosmetic deformity.

The most widely used classification divides JLS into five general types: circumscribed morphea (CM), linear scleroderma, generalized morphea (GM), pansclerotic morphea and the new mixed subtype where a combination of two or more of the previous subtypes is present.

**Circumscribed morphea** (CM) is characterized by oval or round circumscribed areas of induration surrounded by a violaceous halo.

When there are four or more plaques with individual plaques that are larger than 3 cm and they become confluent involving at least two out of seven anatomic sites (head-neck, right upper extremity, left upper extremity, right lower extremity, left lower extremity, anterior trunk, posterior trunk) it is called **Generalized Morphea (GM)**.

**Linear scleroderma**, the most common subtype in children and adolescents, is characterized by one or more linear streaks that can extend through the dermis, subcutaneous tissue, and muscle to the underlying bone, causing significant deformities. The upper or lower extremities can be affected but also the face or scalp, as in the *en coup de sabre *variety (ECDS). The Parry Romberg syndrome (PRS), characterized by hemifacial atrophy of the skin and tissue below the forehead, with mild or absent involvement of the superficial skin is considered the severe end of the spectrum of ECDS and for this reason is included in subtype of linear scleroderma

**Pansclerotic morphea**, an extremely rare but severe subtype, is characterized by generalized full-thickness involvement of the skin of the trunk, extremities, face and scalp with sparing of the fingertips and toes.

A recent multinational study reported that almost one fourth of the patients present extra-cutaneous manifestations such as arthritis, neurological findings, associated autoimmune conditions or ocular abnormalities. Antinuclear antibodies (ANA) are present in more than 40% of patients with JLS.

The management of JLS is challenging and the detection of disease activity and progression remains a fundamental problem. Clinical examination is subjective, classical skin scoring methods, utilized in the assessment of systemic sclerosis, cannot be applied. Among the new tools which have been proposed for the assessment of the skin lesions, infrared thermography (IRT), computerized skin score (CSS), ultrasound (US) and magnetic resonance imaging (MRI) are those most frequently used.

Over the years, many treatments have tried for localized scleroderma. Circumscribed morphea generally is of cosmetic concern only, and therefore treatments with potentially significant toxicity are not justified.

When there is a significant risk for disability, such as in linear and deep subtypes, systemic treatment methotrexate (MTX) in combination with corticosteroids should be considered.

## Disclosure of interest

None declared.

